# Sensory hypo-excitability in a rat model of fetal development in Fragile X Syndrome

**DOI:** 10.1038/srep30769

**Published:** 2016-07-28

**Authors:** Julia Berzhanskaya, Marnie A. Phillips, Jing Shen, Matthew T. Colonnese

**Affiliations:** 1 College of Mechanical and Electronic Engineering, Northwest A&F University, Washington, DC 20052, United States

## Abstract

Fragile X syndrome (FXS) is characterized by sensory hyper-sensitivity, and animal models suggest that neuronal hyper-excitability contributes to this phenotype. To understand how sensory dysfunction develops in FXS, we used the rat model (FMR-KO) to quantify the maturation of cortical visual responses from the onset of responsiveness prior to eye-opening, through age equivalents of human juveniles. Rather than hyper-excitability, visual responses before eye-opening had reduced spike rates and an absence of early gamma oscillations, a marker for normal thalamic function at this age. Despite early hypo-excitability, the developmental trajectory of visual responses in FMR-KO rats was normal, and showed the expected loss of visually evoked bursting at the same age as wild-type, two days before eye-opening. At later ages, during the third and fourth post-natal weeks, signs of mild hyper-excitability emerged. These included an increase in the visually-evoked firing of regular spiking, presumptive excitatory, neurons, and a reduced firing of fast-spiking, presumptive inhibitory, neurons. Our results show that early network changes in the FMR-KO rat arise at ages equivalent to fetal humans and have consequences for excitability that are opposite those found in adults. This suggests identification and treatment should begin early, and be tailored in an age-appropriate manner.

Fragile X syndrome results from decreased expression of the Fragile X mental retardation protein (FMRP), a translational repressor that regulates hundreds of genes, many of them synaptic^1^. FMRP expression is high in the cortex during fetal gestation^2^,^3^, suggesting neural consequences of its absence begin in the womb. How FMRP hypo-expression transforms early cortical synapses, circuits and networks, and how these changes in turn cause the later sensory and cognitive deficits associated with FXS, are still unclear. Identifying the earliest altered circuit function in developing animal models of FXS is an important step toward elucidating mechanism, and developing treatments not only for FXS^4^, but also for non-syndromic autism spectrum disorders (ASD), as FMRP target genes comprise 14 of 27 high-risk genes for ASD^5^.

Mouse knockouts of FMRP show multiple sensory hyper-excitability phenotypes including increased auditory seizure susceptibility^6^ and exaggerated auditory and somatosensory responses^7^,^8^,^9^. A large number of cellular and synaptic defects in these mice, including enhanced protein synthesis-independent long-term depression^10^, impaired interneuron function and density^11^,^12^, disrupted chloride homeostasis^13^,^14^, and misregularion of ion channels^9^,^15^, have suggested that hyper-excitability is a primary contributor to FXS phenotypes^16^. At what point in development this hyper-excitability develops, or even whether hyper-excitability is a characteristic feature of FXS in early development, as it is in the mature brain, is poorly understood. Many early phenotypes of FMRP deletion reverse during later development^14^,^17^,^18^,^19^, while others have delayed onset^20^, suggesting that the effects of FMRP deletion may be age specific.

While FMR-KO mice are an important model of FXS, their small size and fragility make them a poor model of *in vivo* human cortical activity development. Parallels between developing sensory-evoked electrical activity in the unanesthetized mouse and human patients have not been described as they have for the rat^21^. In previous collaborative studies we have used surface electroencephalogram (EEG) to measure spontaneous and sensory-evoked responses in pre-term infants^21^,^22^, and compared these to extracellular depth recordings in developing rat pups *in vivo* using similar protocols and state monitoring^23^,^24^. In terms of spontaneous and evoked cortical activity, rat visual cortex from P4 to eye-opening was found to equate with the late second and third trimesters of human occipital cortical development. During this developmental period sensory stimulation in preterm infants produces very large evoked responses with nested ‘rapid’ oscillations that exactly resemble the ‘delta-brush’ pattern observed in spontaneous EEG activity^21^. Sensory evoked delta-brushes are also observed in rats (where they are called ‘spindle-bursts’). They are evoked by whisker or paw stimulation during the first postnatal week in somatosensory and motor cortex, and by visual stimulation during the second postnatal week in visual cortex^21^,^25^. Somatosensory and visual stimuli also evoke an ‘early-gamma oscillation’ (EGO) in rats that precedes the delta-brush. EGOs occur in response to spatially limited sensory inputs, such as a single whisker^26^ or single digit^27^ stimulation. EGOs are generated in thalamus, and their cortical occurrence is limited to superficial layers. As such they cannot be observed with surface electrodes^24^, so it is not surprising that they have not been observed in the evoked responses of humans. The ability to evoke delta-brush oscillations, however, ends abruptly 2–4 weeks before term in humans^22^,^24^, and in a region specific manner in rats (P8 in barrel cortex; P12 in visual^24^). In this way, cortical activity trajectories in both species are specific for the timeline of development of each sensory system.

Recent production of FMR-KO rats^28^ has allowed us to apply this extensive characterization of normal cortical development to the investigation of early activity patterns relevant to human health. Behavioral and anatomical phenotypes of juvenile FMR-KO rats have been described. They display novel social interaction phenotypes and perseverative chewing not observed in mouse models of the disease^28^. Adult FMR-KO rats show disrupted cortical processing of auditory stimuli^29^, recapitulate multiple hippocampal cellular and synaptic deficits, and show novel hippocampal dependent memory defects^30^, suggesting they are a good model for FXS. Juvenile FMR-KO rats have defective cortical state regularion that begins at ages equivalent to human birth^31^. Despite largely normal patterns of spontaneous activity during the first two post-natal weeks, normal synchronization of firing in deep layers of FMR-KO cortex fails to develop, suggesting that subtle defects of cortical activity may be present in these young animals. Because visual responses can evoke defined activity patterns, including EGOs, and potentially provide a more robust assay in human patients than spontaneous activity, we examined responses to luminance changes in this same population of FMR-KO rats. To understand the age dependence of these findings we investigated visual responses in the rat from the developmental equivalents of pre-term human infants through early childhood, when FXS related symptoms begin to appear.

## Results

We measured the development of cortical visual responses to 100 ms whole-field light flashes in head-fixed un-anaesthetized rats. 30 minute blocks of visual stimulation were acquired before acquiring spontaneous activity in the dark, the analysis of which is published^31^. The data were collected in two separate experiments. In the first (Exp1), exploratory series, males and females from control wild-type litters were compared to males and females from separate FMR-KO litters. In the second (Exp2), confirmatory series, male wild-type controls were compared with FMR-KO male littermates recorded on the same or adjacent days for time periods identified by the exploratory series as critically important for spontaneous or evoked activity.

### Visual responses in infant FMR-KO rats are hypo-excitable and lack early gamma oscillations

**Figure 1:**
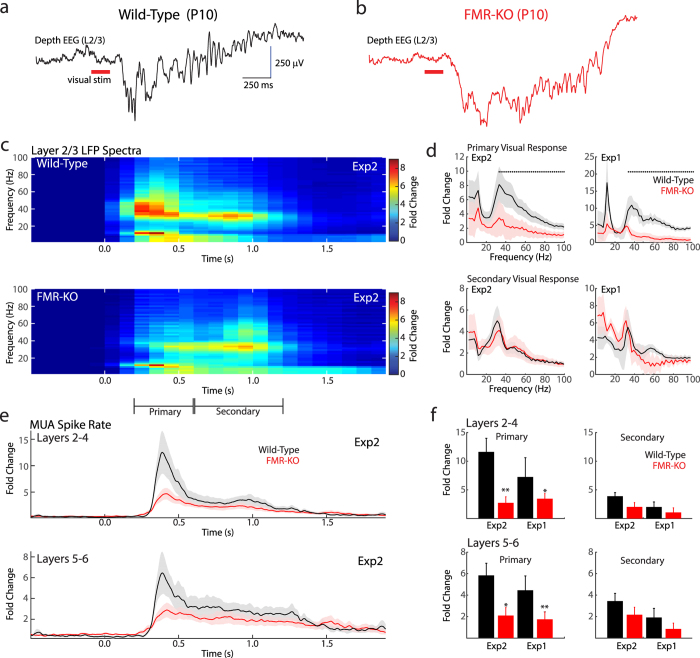
Infant FMR-KO rats lack early gamma oscillations. (**a**) Representative depth EEG (L2/3) response to visual stimulus in a P10 wild-type animal. (**b**) Representative trace for a P10 FMR-KO. (**c**) Population mean (P9–11) peri-stimulus spectrogram of fold increase in dEEG power over baseline (1 s prior to stim) following visual stimulation of wild-type rats (above) and littermate FMR-KO rats (below) for the blinded, confirmatory experiment (Exp2). X-axis is time relative to stimulus onset. Visual responses consist of an initial, ‘primary’, response with nested early gamma oscillations and a ‘secondary’ response with embedded spindle burst (20–30 Hz oscillation). (**d**) Population mean of the peak increase at each frequency for the primary visual response (above) and secondary response (below). Results for Exp2 are in the left column, and for the not-blind exploratory Exp1 on the right. Dots show frequencies with significant difference between groups. (**e**) Population mean peri-stimulus time histogram for visually evoked multi-unit activity in superficial layers (L2–4, above) and deep layers (L5–6, below) for Exp2. Y-axis shows fold increase in firing rate relative to baseline. (**f**) Population mean multi-unit spike rate fold-increases for both experiments during primary (left) and secondary (right) visual responses in superficial (top) and deep (bottom) layers. *p < 0.05; **p < 0.01 by Wilcoxon rank sum. Bars and shading are SEM for all panels.

Visual responses develop before eye-opening and can be evoked in rats directly through the closed eye-lids^24^. These early visual responses reveal the full range of potential network activities in early visual cortex with greater precision than spontaneous activity. Each visual stimulation results in a stereotyped progression of immature oscillations similar to that observed in somatosensory cortex^21^,^26^,^27^. The initial or ‘primary’ response consists of a slow negative potential with nested ‘early gamma oscillations’ (EGOs) driven by thalamic oscillations^32^ (Fig. 1a). EGOs are in turn followed by a secondary response consisting of an oscillation called a spindle-burst, which is 8–30 Hz activity that involves the thalamocortical loop^26^. Spindle-bursts are the major component of the human neonate’s visual response before gestational week 34^24^.

We examined the structure of visual responses at P8–11 in 12 wild-type (5 from Experiment 1, 7 from Exp2) and 16 FMR-KO rats (7 Exp1, 9 Exp2). The visual response in both genotypes consisted of large amplitude negative slow waves that contained faster oscillations and prominent spiking (Fig. 1a,b). Quantitative analysis of the frequency components of the layer 2/3 depth EEG (dEEG) (local field potential) revealed that FMR-KO rats from both Exp1 and Exp2 lack prominent EGOs during the initial response (Fig. 1c only Exp2 shown) but contain normal spindle-burst oscillations. Fold increase in dEEG spectral power over baseline (1 s prior to stim) during the period of EGOs (200–600 ms after visual stimulation) showed significant reduction at all frequencies above 29 Hz (Exp2) or 33 Hz (Exp1) (Fig. 1d, permutation analysis, p < 0.05). Lower frequencies, which result from the large evoked negative wave, were also reduced, though not significantly, in FMR-KO rats. In contrast, during the period of spindle-bursts (600–1200 ms after stimulation), there were no significant differences in either experiment between groups for fold power increase (Fig. 1d). Thus evoked EGOs, but not spindle-bursts, are disrupted in FMR-KO rats.

We evaluated evoked multi-unit spiking responses from electrode contacts located in superficial (L2–4) and deep (L5–6) layers. For both layers, evoked fold-change in firing rate over baseline (1 s prior to stim) were lower in FMR-KO animals specifically during the initial response, but were not different during the spindle-burst period (Fig. 1e Exp2 shown). In wild-type animals, the mean peak spike rate increase in superficial layers 200–600 ms after stimulation was 11.6 ± 2.4 fold over baseline for Exp2, and 7.3 ± 3.6 for Exp1 (Fig. 1f). For FMR-KO animals mean peak spike rate increase was 2.7 ± 0.4 fold for Exp2, and 3.5 ± 0.5 for Exp1 (Wilcoxon Rank Sum, Exp2 p = 0.0012; Exp1 p = 0.041). During the spindle-burst period (600–1200 ms) mean peak spike rate increases were 3.9 ± 0.6 (Exp2) and 2.0 ± 0.9 (Exp1) fold for wild-type, but 2.0 ± 0.3 (Exp2) and 1.1 ± 0.4 (Exp1) for FMR-KO (Exp2 p = 0.112; Exp1 p = 0.317). For deep layers mean peak spike rate increases were 5.8 ± 1.1 (Exp2) and 4.4 ± 1.3 (Exp1) fold for wild-type, and 2.1 ± 0.6 (Exp2) and 1.7 ± 0.6 (Exp1) fold for FMR-KO (Exp2 p = 0.014; Exp1 p = 0.005) during the EGO period, and 3.4 ± 0.7 (Exp2) and 1.9 ± 0.8 (Exp1) fold for wild-type and 2.2 ± 0.6 (Exp2) and 0.6 ± 0.5 (Exp1) fold for FMR-KO (Exp2 p = 0.295; Exp1 p = 0.096) during the spindle-burst period. Thus in two separate experiments we observed that both evoked spike-rates and dEEG frequencies are specifically reduced in FMR-KO rats during EGOs, but not spindle-bursts. Spontaneous spike rates are not altered by FMR-KO at these ages^31^, suggesting the reduced evoked firing rates are the result of decreased firing following stimulation.

### Normal developmental down-regulation of visual responses in FMR-KO rats

**Figure 2:**
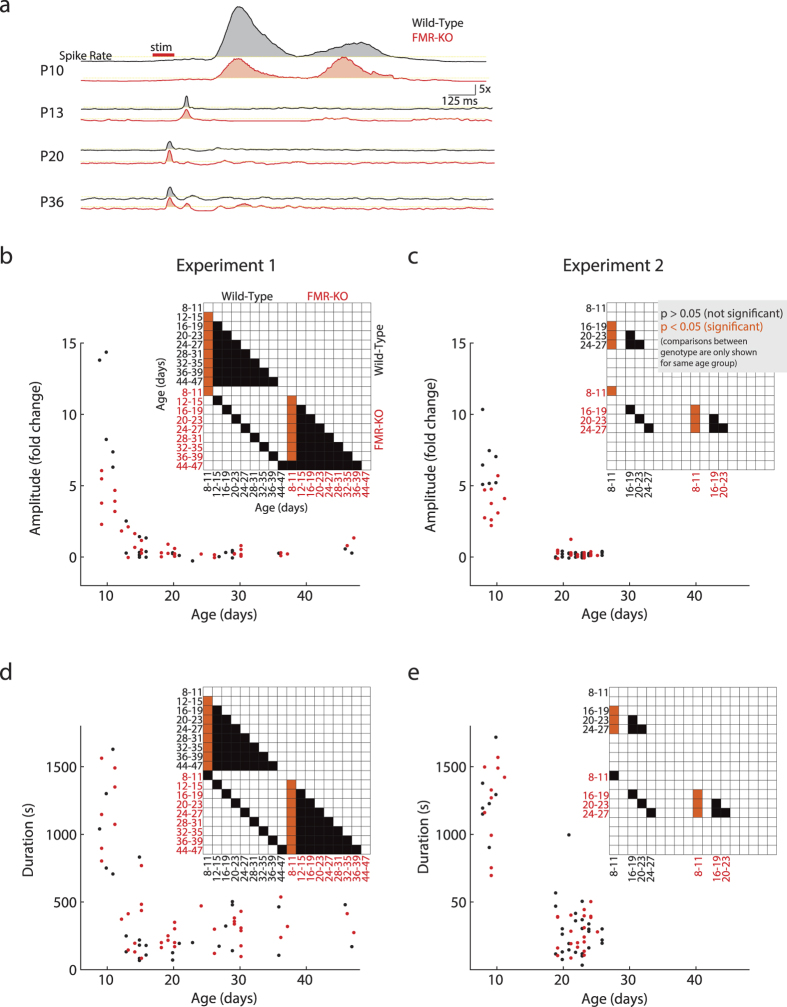
Developmental sharpening of visual responses is not delayed in FMR-KO rats. (**a**) Representative post-stimulus time histograms show fold-increase in multi-unit activity for littermates (Exp2). Note large drop in amplitude (but sharper tuning) of evoked response in both groups between P10 and P12. (**b**) Development of visual response amplitudes for Exp1. Integrated fold-change in multi-unit spike-rates (all layers) is graphed by age of animal. Matrix of pair-wise significance differences (Tukey post-hoc) for amplitude four-day means is shown as inset. Comparisons between different ages within genotype are shown to demonstrate that the developmental shift between P11 and P12 occurs in both groups. Comparisons between genotypes for the same ages (eg. P8–9 WT vs P8–9 KO) are shown to demonstrate that amplitude is affected by FMR-KO specifically during the early developmental period (P8–11). Comparisons between WT and KO at different ages are not shown. Orange = p < 0.05; black = p > 0.05; white = comparison not shown for clarity. (**c**) Development of amplitudes for Exp2. (**d**) Development of visual response duration for Exp1. Note that duration P8–11 is not different between genotypes. (**e**) Development of duration for Exp2.

In normal rats, visual responses to flash stimuli are reduced in both total amplitude and duration as part of a switch in the thalamocortical network properties that occurs just before (P12) eye-opening (P14). A similar switch occurs just before (gestational week 35) normal term (week 38–40) in humans^24^. To determine if FMR-KO rats demonstrate developmental delay of this switch we measured the amplitude (integrated fold-change in firing rate) and duration (total duration of significantly elevated firing rates) for each animal as function of age (Fig. 2). In Exp1, which contained near daily recordings, both genotypes demonstrated the normal down-regulation of visual response amplitude and duration between P11 and P12 (Fig. 2b,d). Consistent with the hypo-excitability of the primary response in FMR-KO animals, P8–11 knock-outs from both Exp1 and Exp2 had reduced visually evoked spike rates. mANOVA using four day intervals in Exp1 revealed significant effects of age (df = 8, F = 42.77, p = 10^−23^), group (df = 1,F = 4.09, p = 0.048), and an interaction between the two (df = 8,F = 9.71, p = 10^−5^). Because recordings in Exp2 were targeted to early and juvenile periods, the 4-day periods were restricted to P8–11, P16–19, P20–23, and P24–27, but this group also showed significant effects of age (df = 3, F = 205.29, p = 10^−34^), group (df = 1, F = 13.61, p = 0.0004), and interaction (df = 3, F = 20.39, p = 10^−9^). Tukey post-hoc analysis demonstrated two clear developmental periods (P8–11 and P12+) for both genotypes, with significant differences between wild-type and FMR-KO limited to the P8–11 age for both Exp1 and Exp2 (Fig. 2b,c). Duration showed a similar developmental pattern, but there were no differences between wild-type and FMR-KO at any age (Fig. 2d,e; Exp1 age (F = 18.77, p = 10^−14^), group (F = 1.28, p = 0.26), interaction (F = 0.26, p = 0.98); Exp2 age (F = 101.83, p = 10^−25^), group (F = 0.1, p = 0.76), interaction (F = 0.48, p = 0.70)). Tukey-post hoc analysis again revealed two age groups in both wild-type and FMR-KO, but there was no difference between genotypes at P8–11 for duration.

These data show that visual response hypo-excitability in FMR-KO rats is limited to the period of immature oscillations, and that the switch to mature response patterns is not significantly delayed in FMR-KO rats.

### Altered single-unit visual responses in juvenile FMR-KO rats

While total amplitude and response duration of multi-unit responses were not altered in FMR-KO rats during the third and fourth postnatal weeks, these gross measures can obscure other potential changes. We therefore examined visual responses in juvenile (P19–24) rats. For consistency during this rapidly changing developmental period^33^ we examined the animals in Exp2 for which we had paired littermates at the same ages (Exp2, n = 22 wild-type, n = 21 FMR-KO).

**Figure 3:**
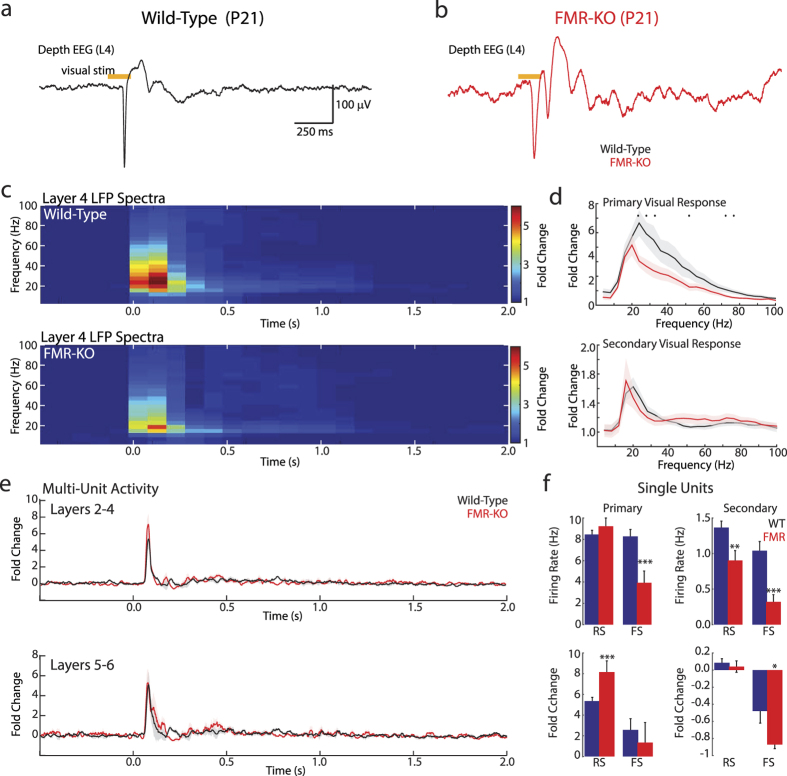
Juvenile FMR-KO rats have hyper-excitable visual responses. (**a**) Representative dEEG (L4) response to 100 ms light flash of a wild-type animal. (**b**) Representative response of FMR-KO rat. (**c**) Population mean (P19–24) time-spectrogram of fold-increase over baseline (1 s pre-stimulus) for L4 dEEG for wild-type (above) and FMR-KO (below). All juvenile analysis from Exp2. Time axis is aligned to onset of visual stimulus. Visual responses are divided into primary (0–250 ms) and secondary (300–1200 ms) responses. (**d**) Population mean of spectral fold-increase over baseline for primary (top) and secondary (bottom) L4 dEEG. Spectral bins with significant difference (p < 0.05) between wild-type and FMR-KO are shown by black dots. (**e**) Population mean multi-unit peri-stimulus time histogram of fold increase over baseline in firing rates for superficial (L2–4, top) and deep (L5–6, bottom) neurons. (**f**) Visual response characteristics of single-units. Firing rate change measured by absolute firing-rate (top) during primary response (left) and secondary response (right), and by fold-increase over baseline (2 s pre-stimulus; bottom) for the same periods. Regular spiking (RS; excitatory) and fast-spiking (FS; inhibitory) neurons are graphed separately. ***p < 0.001, *p < 0.05 by t-test.

The mature visual response to whole field light flashes consisted of a large primary (0–200 ms post-stimulus) response composed of a fast, sharp negative dEEG potential that was largest in layer 4 and a rapid increase in firing rates in all layers (Fig. 3a,b). We observed a more variable secondary (300–900 ms) response, which included negative and positive dEEG potentials and slight increases in firing rate. Like the mutant mouse^34^, the amplitude of the initial negative deflection of the L4 dEEG primary response was not significantly different between groups (−370 ± 30 μV wild-type vs −310 ± 30 μV FMR-KO; p = 0.19). However, spectral analysis revealed that the FMR-KO primary response contains less visually evoked power in frequencies above 20 Hz (Fig. 3c,d). Permutation analysis (p < 0.05) showed significant reductions only in spectral bins with centers at 24, 28, 32, 52, 76, and 80 Hz. Because the mature evoked potential does not contain strong oscillations, the altered frequencies in FMR-KO likely reflect changes in the spectral composition of the primary negative and positive fluctuations.

Analysis of the multi-unit peri-stimulus time histogram revealed no significant differences between groups in superficial or deep layers (Fig. 3e). The peak MUA spike rate increase for superficial layers during the primary visual response was 5.9 ± 0.8 fold for wild-type and 7.3 ± 1.2 fold for FMR-KO (t-test, p = 0.42); mean spike rate increase for the secondary response was 0.4 ± 0.1 fold for wild-type and 0.4 ± 0.1 fold for FMR-KO (p = 0.95). In deep layers primary responses were 5.2 ± 0.7 fold for wild-type and 5.7 ± 1.3 fold for FMR-KO (p = 0.74), while secondary responses were 0.3 ± 0.1 fold for wild-type and 0.4 ± 0.2 fold for FMR-KO (p = 0.61). The data are not shown as separate bar graphs, but are reflected in the peri-stimulus time histogram (Fig. 3e).

Multi-unit activity as a mix of neuronal types can obscure changes in firing rates. We therefore examined visual responses of single units isolated by spike sorting. The units were divided into two groups based on Peak-Trough delay and repolarization time: Regular Spiking, presumptive excitatory neurons (n = 253 wild-type, 186 FMR-KO) and fast-spiking, presumptive inhibitory neurons (n = 75 wild-type, n = 36 FMR-KO). The spontaneous activity of these neurons has been previously reported^31^. In the dark, firing rates of Regular spiking, but not fast-spiking neurons, is significantly reduced in this population of neurons; an effect caused by the increased prevalence of the aroused/desynchronized cortical state. In the present data, firing rate distributions were not normally distributed (Andersen-Darling test, p < 0.05) and so are presented as median ± standard error of the median and analyzed using non-parametric tests. The lower prevalence of fast-spiking neurons means that fast-spiking neurons require an effect size 2.09 fold that of regular-spiking neurons to achieve similar power^35^. The effect size required for 0.8 power was 0.28 for regular-spiking and 0.59 for fast-spiking neurons. Firing during the baseline period (2 s prior to stimulus) was similar to that observed spontaneously in the dark. Median baseline firing-rate of regular spiking neurons was 1.30 ± 0.08 Hz in wild-type and 0.82 ± 0.18 Hz in FMR-KO (Wilcoxon rank-sum (all subsequent tests), p = 0.012, effect-size (r) = 0.134); for fast-spiking neurons baseline firing-rate was 2.13 ± 0.37 Hz for wild-type and 1.11 ± 1.79 Hz in FMR-KO (p = 0.335, r = 0.102)(data not shown). In wild-type, 39% of regular-spiking and 51% of fast-spiking neurons had peak evoked spike rates greater than 3 standard deviations above baseline. For FMR-KO the response rate was 42% for regular spiking and 65% for fast-spiking. Because it is unclear whether absolute or relative stimulus-induced spike rates are more important for behavior, we quantified visually evoked change in spike rates as both absolute firing rate and fold-change over baseline ((FR-baseline)/baseline) (Fig. 3f). For the primary response, regular-spiking neurons had similar peak firing rates (8.45 ± 0.41 Hz wild-type vs 9.23 ± 0.77 Hz FMR-KO, p = 0.57, r = 0.030) but because baseline spike rates are lower in FMR-KO the stimulus-induced increase in firing was greater in FMR-KO rats (5.36 ± 0.36 fold wild-type vs. 8.15 ± 1.09 fold FMR-KO, p = 0.00055, r = 0.185)). Fast-spiking neurons of FMR-KO rats showed reduced absolute firing during the primary response (8.27 ± 0.69 Hz wild-type vs 3.92 ± 0.63 Hz FMR-KO, p = 0.0038, r = 0.305), but relative rates were not significantly changed (2.60 ± 1.07 fold wild-type vs. 1.35 ± 1.94 fold FMR-KO, p = 0.21, r = 0.133, β = 0.71). During the secondary response regular-spiking neurons showed very little change in spiking from baseline. As a result FMR-KO firing rates were significantly lower than wild-type (1.36 ± 0.09 Hz wild-type vs 0.89 ± 0.15 Hz FMR-KO, p = 0.0047, r = 0.151) but fold-increase over baseline was similar (0.09 ± 0.05 fold wild-type vs 0.04 ± 0.07 fold FMR-KO, p = 0.52, r = 0.034). Fast-spiking neurons showed an unexpected pattern of reduced firing during the secondary response that was exacerbated in FMR-KO pups (absolute spike-rates: 1.04 ± 0.13 Hz wild-type vs 0.32 ± 0.08 Hz FMR-KO, p = 0.0001, r = 0.411; fold change: (−0.48 ± 0.14 fold wild-type vs −0.87 ± 0.05 fold FMR-KO, p = 0.017, r  =  0.273)).

In sum, single unit responses indicate a complex group of changes in neuronal behavior that is consistent with mild hyper-excitability of visual responses in juvenile FMR-KO rats. Regular spiking neurons have increased visual responsiveness as a result of decreased spontaneous firing, consistent with the increased prevalence of the aroused cortical state in the animals^31^,^36^. Fast-spiking neurons showed a trend toward reduced firing rates in FMR-KO rats relative to wild-type following visual stimulation, though the low-incidence of this neural population and their large variability, particularly in FMR-KO rats, means that more targeted methods are required to fully elucidate their regulation by FMRP.

## Discussion

We have conducted a systematic study of the development of light-evoked activity in the visual cortex of the FMR-KO rat. We used simple luminance stimuli because they can evoke responses through closed eye-lids in rats and humans^24^. We found that while the gross trajectory of visual response development is largely intact in FMR-KO rats, there exist multiple age-specific changes in circuit function that may contribute to FXS dysfunction. Most prominently, visual responses before eye-opening, during the period of sensory-evoked bursting and dense-synchronous cortical firing^37^,^38^,^39^, show hypo-excitability in the form of reduced γ-power and neuronal firing during the initial visual response. This hypo-excitability was not present at later ages, after immature evoked oscillations disappear. Instead, juvenile visual responses became mildly hyper-excitable, with increased firing of regular-spiking (excitatory neurons) and decreased firing of fast-spiking (inhibitory) neurons. Our results show that the cellular, synaptic and circuit defects caused by elimination of FMRP have age-specific consequences for emergent network activity. In particular these data suggest that sensory hyper-excitability is not present from the beginning, but rather emerges only after hypo-excitability of the earliest sensory response.

dEEG patterns of visually evoked and spontaneous activity in the visual cortex of rats P4-P11 resemble the EEGs of preterm infants of gestational age 28–34 weeks^21^,^40^. While the frequency, occurrence, or duration of spontaneous activity is not altered in these FMR-KO rats^31^, their visual responses are affected. The primary change we observed in mutants during this ‘pre-term period’ is an absence of ‘early gamma oscillations’. EGOs in visual cortex are observed primarily as a component of the initial visual response, which is the direct thalamic input evoked by light^24^, but are not observed during spontaneous activity^41^, even though this activity is also retinally derived. EGOs are the result of the feed-forward propagation of oscillatory thalamic firing, and not of cortical interneurons as in adult gamma^32^. Cortical inhibition does contribute to EGOs by preventing runaway activity, and inhibition is weakened in neonatal FMR-KO cortex as a result of increased Na^+^-K^+^-Cl^−^ cotransporter (NKCC1) expression^14^, suggesting a potential mechanism for EGO elimination. However reduced GABA_A_ function should increase firing during early gamma^42^, not decrease it as we observed, suggesting additional circuit changes are causal. Loss of the thalamic gamma oscillator, and weaker or less refined thalamocortical input are likely contributors to the loss of EGOs. Thalamus is the region with the largest reduction of GABA_A_ receptor binding in FXS patients^43^. Fragile X granules are expressed in thalamocortical axons in mouse (but not in thalamic interneurons or reticular neurons)^44^ and glutamatergic synaptic development at thalamocortical synapses is delayed in FMR-KO mice^45^. By the juvenile period however, thalamocortical synaptic strength is normalized and local cortical hyperconnectivity emerges^46^, which may explain the transition between hypo and hyper excitability we observed.

Spindle-burst oscillations, which compose the secondary visual response and are the oscillations driven by retinal waves^21^, were not affected in the FMR-KO^31^. Why EGOs but not spindle-bursts are affected is difficult to explain, as both require retinal activity via the thalamus^21^. One possibility is that spindle-bursts involve the entire cortical circuit while EGOs are limited to the input layer^24^,^26^. Thus during spindle-bursts, amplification in recurrent cortical and thalamocortical networks may compensate for weak thalamic input. In addition, EGOs, but not spindle-bursts, require activation of a small topographic region. Thus disrupted topography of retinal or thalamic connections could reduce gamma, but not spindle-burst, power.

With the exception of the lack of EGOs, the developmental trajectory of visual responses was normal in FMR-KO animals: the developmental switch in visual responsiveness–which includes elimination of immature bursting, acquisition of the mature visual evoked potential and sparsification of responses^24^,^38^–occurred normally between P11 and P13. This is consistent with FMR-KO mice, which undergo a similar, though incomplete, switch in somatosensory cortex at equivalent ages^20^,^39^. This switch is part of a larger developmental change in cortical network properties linked to the onset of the cortical ‘active’ state^37^,^38^,^39^ that allows the modulation of cortical activity by arousal^33^,^47^. Remarkably, the onset of cortical active states is not delayed *in vivo*, although it is delayed in organotypic cultures^48^.

After this switch, during the third and fourth post-natal weeks, visual responses as measured by multi-unit activity and evoked potential amplitudes were normal in FMR-KO rats, consistent with findings in mice^34^. Close examination of single-unit responses, separated into presumptive neuron classes, indicate a subtle hyper-excitability to visual stimuli. Why significant changes were not observed in the MUA response is not clear, but could result from the increased noise inherent in including fast-spiking neurons (which are not affected but have high baseline firing rates) and excluding poorly sorted spikes. Hyper-excitability to pure-tones is observed in anesthetized FMR-KO mouse auditory cortex^49^, while anesthetized FMR-KO rats actually show decreased firing to human speech and tones in ventral auditory field^29^. Cortical whisker responses are elevated^8^, and forepaw-evoked action potential firing more than triples in anesthetized FMR-KO mice as a result of defective voltage-gated calcium channel activity^9^. Our visual stimuli were optimized to track the early development of visual responses, and the rats were not trained in a task that would engage visual processing, so how the changes we observed translate into visual processing defects is unknown. Hyper-excitability and processing defects are greater for audition than for vision in FXS^50^,^51^, and visual processing deficits in FXS are limited to the dorsal stream^52^,^53^ so the mild hyper-excitability observed here is expected. In this same population we observed persistent activation of visual cortex during quiescent periods^31^, and many of the cortical network changes observed in FMR-KO mice involve cortical state regularion^20^,^48^,^54^. The fact that incresed regular spiking neuron responsiveness is the result of decreased relative baseline activity, leading to greater total signal to noise for this population, suggests that the changes we observe are the result of cortical state changes rather than pure hyper-excitability in the visual path. The sensory hyper-excitability described in mice could also be due largely to decreased susceptibility to anesthesia^20^. Together these results suggest that the circuits affected by loss of FMRP strongly involve the regulation of cortical state, and therefore attention and arousal, more significantly than circuits of direct sensory transmission, at least in the juvenile and mature brain.

Overall, our data on neonatal pups show that cortical circuit changes caused by FMR-KO can modify activity at very young ages, and suggest that diagnosis and treatment could be informed by EEG monitoring of neonates. Most interestingly the changes caused by FMR-KO are age specific, and so treatments must be targeted accordingly. While superficially the absence of EGOs in the neonate suggests a possible biomarker, sensory evoked EGOs have so far not been detected by conventional EEG^22^,^24^, probably because even in the rat, they are largely restricted to layer 4 and do not propagate to the surface^24^,^42^.

## Methods

### Subjects

All experiments were conducted with approval from The George Washington University School of Medicine and Health Sciences Institutional Animal Care and Use Committee, in accordance with the *Guide for the Care and Use of Laboratory Animals* (8^th^ Edition, National Academies Press). Experiment design, recording methodology, and analysis was as reported^31^ Sprague-Dawley FMR-KO rats were acquired from SAGE Labs (St Louis MO). Experiments were conducted in two separate series. In the 1^st^ series FMR^−/−^ and FMR^y/−^ pups were either shipped at P4 directly from Sage Labs or bred in the local animal facility. Sprague-Dawley rats acquired from Hilltop Lab Animals (Scottdale, PA) in a similar manner were used as wild-type. Because whole litters were KO or WT, female and male animals were used. In the second series, to be able to compare littermates, only male mutant and wild-type littermates were examined. These were obtained by crossing FMR^+/−^ females with wild-type males. Animals were identified and sexed by genotyping for the presence of the mutant or wild-type gene and presence of the Y chromosome (Transnetyx, Cordova TN). For the second series, experiments and analysis were performed blind to genotype on both a wild-type and an FMR-KO littermate on the same or subsequent day.

### Surgery and Recordings

For installation of the head-fixation apparatus animals were given sub-cutaneous carprofen injections (5 mg/kg weight) and anesthetized with 2–3% isoflurane. Adequate depth of anesthesia was verified by toe pinch and breathing rate. Resection of the scalp was made using aseptic technique, the skull was cleaned of connective tissue, and electrode locations were marked on the skull. A head fixation bar and vertical frontal mounting pole were attached to the skull with dental cement, leaving the recording window open.

For recording the animal was placed in a modified stereotaxic apparatus that attached to the headgear under isoflurane anesthesia. Body restraint was provided by soft sterile cloth lined tube. Body temperature (measured under the chest) was maintained between 32C and 36C via an electric heating pad placed under the tube. Monocular visual cortex was targeted with the following coordinates: 0–0.5 mm anterior from *lambda* and 2.5 (p4–5), 2.8 (p9–11), or 3.0–3.5 (p13+) lateral. An Ag/AgCl wire was placed over right frontal cortex (~1 mm anterior and 3 mm lateral to *bregma*) as ground. The skull over frontal pole (for ground insertion) and over visual cortex was thinned until transparent. The final layer of bone was chipped until producing a craniotomy 100–200 um diameter. In older animals the dura was resected. A stainless steel wire (100 um diameter) was placed in the facial muscle for electromyography (EMG), and a piezoelectric motion detector was placed under the animal holding tube.

Neural activity was recorded using Neuronexus (Ann Arbor, MI) 32 channel ‘Poly2’ probes, consisting of two parallel rows of 16 sites, separated by 50 μm. Probes were positioned approximately radially to the cortical layers using a micro manipulator. All electrodes were coated in DiI (Sigma, Saint Louis MO) to allow post-experiment penetration localization. Electrical signals were collected using Neuralynx (Bozeman, MT) Digital Lynx S hardware with Cheetah (v5.5) software. dEEG signals were band-pass filtered between 0.1 Hz to 9 kHz, and digitized at 32 kHz. All recordings were referenced to the bottom electrode to eliminate large slow potential variations and common mode noise. Spikes were extracted by threshold crossing of −40 μV in the 600 Hz −9 kHz band-pass signal, saved as 1 ms, 32 point waveforms for all 4 contacts in a tetrode. Each of the 7 tetrodes per shaft consisted of 4-adjacent contacts, two on each row, with no overlap of contacts between tetrodes.

Full field visual stimulation was delivered using a white light emitting diode positioned 2–5 mm from the contralateral eye. Visual stimulation consisted of 100 ms flashes given at 0.05 Hz. Before the unanesthetized experiments, dEEG responses were measured at multiple illuminance levels between 10 and 100 lux under 1% isoflurane. A single value that produced between 50–75% of the maximum dEEG amplitude was selected for the visual evoked experiments. Recording of visually evoked activity began 20 minutes after the animals began spontaneous movements (usually about 10 min) after isoflurane was removed.

### Data analysis

Neural signals were imported into Matlab (Mathworks, MA). dEEG was down-sampled to 1 kHz. Layer 4 was identified in each recording as the layer with the earliest negative deflection in the mean visual evoked response. Location relative to the electrode with the highest firing rate (Layer 5a) as well as absolute depth from the cortical surface (at least 350 um) were used to resolve ambiguity. Periods of sleep, defined as the absence of movement or EMG signal for longer than 5 minutes, were removed, as were periods of movement, defined by EMG signal exceeding a manually selected baseline. Approximately 25% of animals expressed periods of 2–4 Hz high-voltage spike-wave complexes, commonly detected in Sprague-Dawley rats^55^. In the first group of experiments this activity was linked to genotype, with 12% of wild-type but 43% of FMR-KO rats displaying spike-wave discharges for longer than 10 s. In the second group of experiments, sharp wave activity was not linked to genotype, and occurred in 24% and 28% of FMR-KO and wild-type animals, respectively. Periods including spike-wave discharges were excluded from analysis by elimination of all 1 minute periods containing dEEG activity >600 μV.

dEEG spectra were obtained by multitaper method using the freely available Chronux package^56^ with taper parameters [3 5]. For light evoked activity P9–11, a 0.5 s sliding (0.1 s steps) multi-taper window was applied −2–5 s relative to the stimulus, and the spectra averaged across 20–40 stimuli. The visual response was calculated as percent increase over mean baseline (−1.2–−0.2 s) power at each frequency. Percent increase for spectra and MUA is calculated as (resp-baseline)/baseline, where resp is the value of interest. Duration of visual responses was calculated from the stimulus averaged MUA spike trains as the number of 10 ms bins in the two seconds following the stimulus with firing rates greater than 3 SD of the baseline (1 s pre-stimulus) firing rate.

### Single Unit Analysis

Putative single unit isolation was done based on spike shapes using the masked EM algorithm (Klustakwik^57^) in Peak, Energy, PCA2 space for all 4 electrodes in each tetrode. Instead of manual selection of clusters for inclusion based on visual inspection of the wave-forms, clusters were further refined based on shape similarity of spikes in a cluster using custom written Matlab code. For each spike in a cluster the mean Euclidian distance from the mean spike waveform 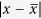 was calculated, where x is the individual spike waveform, 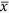 is average spike waveform for the cluster. In other words, for each spike, the total voltage difference remaining after subtraction of that spike’s voltage waveform from the waveform of the mean spike is calculated. The similarity metric is then computed as follows:


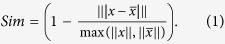


This normalizes the difference to the peak of the larger spike and makes high similarity approach 1 and low similarity approach 0. The mean *Similarity* of all spikes in a cleaned cluster was then used to evaluate clusters as originating from a single unit. Visual inspection of >100 clusters determined a mean *Similarity* of >0.58 to correspond to a human observer’s opinion of a waveform distribution generated by a single neuron. Clusters were included in the analysis with the following minimal measures^58^: *Isolation Distance* >15, *L*_*ratio*_ < 0.5, percentage of spikes with an inter-spike interval below 2 ms <2%, mean *Similarity* >0.58, and number of spikes >60. With this method, 60–80% of all spikes were assigned to good clusters and included in the analysis. Further visual inspection of spike clusters was used to eliminate clusters with average waveforms that were distorted or contained electrical artefacts (<1% of all isolated units). To split clusters into functional neuron classes, we measured peak-valley ratio, peak-valley delay and relative repolarization within 500 ms of peak. Three dimensional hierarchical clustering identified two groups, which could be separated by repolarization threshold alone. Neurons with low repolarization (<0.58) were classified as regular-spiking cells, those above this threshold as fast-spiking. Neurons reported here are the same as previously reported for spontaneous activity^31^.

Single unit evoked firing rates were calculated as percent change ***FR***_***pc***_*** ***= ***(FR***_***resp***_***- FR***_***pre***_)/***FR***_***pre***_ where *FR*_*pre*_ = the mean firing rate in the pre-stimulus period (2 seconds prior) and *FR*_*resp*_ is the firing rate during the period of interest. Longer baseline periods were used for single-unit analysis to more accurately measure spontaneous rates.

### Statistics

Distributions were evaluated for normality using the Anderson-Darling test. Normally distributed data are reported as mean ± standard error of the mean (SEM). Non-normal data are reported as median ± standard error of the median as determined by bootstrapping (1000 iterations). Power and effect size was calculated using the freely distributed software G-power^35^ for non-parametric data using the Wilcoxon rank-sum test. Spectra were examined at each frequency for significant difference using non-parametric permutation tests corrected for multiple comparisons by the method of^59^. All other tests are described in the Results and performed in Matlab. Non-parametric hypothesis tests were applied for comparisons where n < 10.

## Additional Information

**How to cite this article**: Berzhanskaya, J. *et al.* Sensory hypo-excitability in a rat model of fetal development in Fragile X Syndrome. *Sci. Rep.* **6**, 30769; doi: 10.1038/srep30769 (2016).
