# Erratum: Sensory hypo-excitability in a rat model of fetal development in Fragile X Syndrome

**DOI:** 10.1038/srep44515

**Published:** 2017-03-15

**Authors:** Julia Berzhanskaya, Marnie A. Phillips, Jing Shen, Matthew T. Colonnese

Scientific Reports 6: Article number: 3076910.1038/srep30769; published online: 07 28 2016; updated: 03 15 2017

The original HTML version of this Article listed an incorrect Affiliation for all authors. The correct Affiliation is listed below:

Institute for Neuroscience, Department of Pharmacology and Physiology, The George Washington University, Washington DC 20037, United States.

This has now been corrected in the HTML version of this Article; the PDF version was correct at the time of publication.

